# Supplementary material for: Multi‐dataset identification of innovative feature genes and molecular mechanisms in keratoconus
Source: J Cell Mol Med. 2024 Sep 19;28(18):e70079. doi: 10.1111/jcmm.70079 (PMC11412914; doi:10.1111/jcmm.70079)
Supplement: Supplementary file 2 — Material S2. [file JCMM-28-e70079-s002.zip › 7.GSEA analysis/1.ARL11/KEGG_GLYCOSAMINOGLYCAN_BIOSYNTHESIS_CHONDROITIN_SULFATE.html]

Details for gene set KEGG\_GLYCOSAMINOGLYCAN\_BIOSYNTHESIS\_CHONDROITIN\_SULFATE[GSEA]

|  || Dataset | ARL11.group.cls#h\_versus\_l |
| Phenotype | group.cls#h\_versus\_l |
| Upregulated in class | l |
| GeneSet | KEGG\_GLYCOSAMINOGLYCAN\_BIOSYNTHESIS\_CHONDROITIN\_SULFATE |
| Enrichment Score (ES) | -0.5368879 |
| Normalized Enrichment Score (NES) | -1.4335532 |
| Nominal p-value | 0.08906882 |
| FDR q-value | 1.0 |
| FWER p-Value | 0.856 |
Table: GSEA Results Summary

  

Fig 1: Enrichment plot: KEGG\_GLYCOSAMINOGLYCAN\_BIOSYNTHESIS\_CHONDROITIN\_SULFATE      
 Profile of the Running ES Score & Positions of GeneSet Members on the Rank Ordered List

  

| SYMBOL | TITLE | RANK IN GENE LIST | RANK METRIC SCORE | RUNNING ES | CORE ENRICHMENT || 1 | B3GALT6 | na | 11318 | 0.044 | -0.3027 | No |
| 2 | XYLT2 | na | 12163 | 0.035 | -0.3084 | No |
| 3 | B3GAT3 | na | 12696 | 0.030 | -0.3078 | No |
| 4 | CHSY1 | na | 13702 | 0.021 | -0.3254 | No |
| 5 | CHPF | na | 13989 | 0.019 | -0.3236 | No |
| 6 | CHST3 | na | 16308 | 0.002 | -0.3895 | No |
| 7 | CHST14 | na | 16966 | -0.003 | -0.4068 | No |
| 8 | UST | na | 18294 | -0.012 | -0.4384 | No |
| 9 | CHST12 | na | 18671 | -0.015 | -0.4413 | No |
| 10 | B4GALT7 | na | 19329 | -0.019 | -0.4499 | No |
| 11 | DSE | na | 21768 | -0.037 | -0.5001 | No |
| 12 | B3GAT2 | na | 21892 | -0.039 | -0.4830 | No |
| 13 | CHST13 | na | 23765 | -0.059 | -0.5050 | Yes |
| 14 | XYLT1 | na | 24720 | -0.072 | -0.4937 | Yes |
| 15 | CHPF2 | na | 24961 | -0.075 | -0.4601 | Yes |
| 16 | B3GAT1 | na | 25591 | -0.084 | -0.4330 | Yes |
| 17 | CSGALNACT1 | na | 28098 | -0.134 | -0.4334 | Yes |
| 18 | CHST11 | na | 29434 | -0.170 | -0.3805 | Yes |
| 19 | CHSY3 | na | 31075 | -0.223 | -0.3081 | Yes |
| 20 | CHST7 | na | 31454 | -0.237 | -0.1915 | Yes |
| 21 | CHST15 | na | 31919 | -0.257 | -0.0670 | Yes |
| 22 | CSGALNACT2 | na | 32321 | -0.275 | 0.0694 | Yes |
Table: GSEA details [plain text format]

  

Fig 2: KEGG\_GLYCOSAMINOGLYCAN\_BIOSYNTHESIS\_CHONDROITIN\_SULFATE      
 Blue-Pink O' Gram in the Space of the Analyzed GeneSet

  

Fig 3: KEGG\_GLYCOSAMINOGLYCAN\_BIOSYNTHESIS\_CHONDROITIN\_SULFATE: Random ES distribution      
 Gene set null distribution of ES for **KEGG\_GLYCOSAMINOGLYCAN\_BIOSYNTHESIS\_CHONDROITIN\_SULFATE**
